# Dr. A. P. W. Philip on Indigestion

**Published:** 1822-03-01

**Authors:** 


*A Treatise on Indigestion and its Consequences, called Nervous and Bilious Complaints; with Observations on the Organic Diseases, in which they sometimes terminate.* By A. P. W. Philip, M. D. F. R. S. Ed. &c. One vol. 8vo, pp. 391. Second Edition, with some additional Observations. London, 1822.

We cannot inform our readers more concisely of the changes made in the present edition of the above work, than by quoting the following short preface to this edition.

"In revising the following Treatise, the author's only object has been to render it more useful to the practitioner.

"As hardly half a year has elapsed since its publication, any considerable enlargement of it is not to be expected; but he hopes that the present edition will be found in several respects improved. Many observations have been added, and an attempt has been made, in several passages, to explain more fully the principles which led to the treatment recommended in it, the author's confidence in which, he is happy to say, has been strengthened since the appearance of the first edition, by communications from several physicians, to some of whom he has not the honour of being personally known.

"For the sake of hasty readers, who seldom see arrangement in a work where the subject is at all complicated, without numerous divisions and references, the author has introduced a greater number of these, which, to the diligent who bear in mind the plan laid down and feel no difficulty in perceiving how the different parts of the subject arrange themselves under it, are often superfluous, and therefore unwelcome interruptions." *Pref.* xii.

It is impossible to follow the author through every part of the work, and note all the alterations which may be observed in it. They consist of additional observations, and the introduction of more frequent titles, which render it more easy of reference. We shall quote from the additional observations in this edition, the following passage on the combination of the second stage of indigestion and fever. After remarking on the frequency of this combination, and stating his view of the general nature of fever, he observes :—

"Now, in those who labour under the second stage of indigestion, we have seen that some of those parts which greatly sympathize with the stomach, generally suffer most. These, therefore, are the weak parts which most feel the effect of the morbidly increased force of circulation in fever. Their vessels are most apt to suffer distention, producing congestion or inflammation, according as the distention is in larger or smaller vessels.

" The liver, it appears from what has been said, is the part which most frequently suffers by sympathy in the second stage of indigestion. It therefore often happens that, when those labouring under this stage are attacked with fever, a train of symptoms similar to that detailed in page 271, supervenes, the proper treatment of which is essential to that of the fever.

" The principle of the treatment of these symptoms, when they occur in fever, is precisely the same as where they supervene without it, but the actual practice is not altogether so. The fever, in its early stages, by adding to the severity of the inflammatory symptoms, renders more active means necessary. Hence, if the general symptoms do not indicate general loss of blood, a greater local abstraction of it is usually proper, than when no fever but that occasioned by the local affection attends.

" The same observation applies to the use of cathartics. At the commencement of fever the free action of the bowels is particularly beneficial, and by such a state of the liver is rendered doubly so. It is thus that brisk doses of calomel at this period are generally more beneficial than other mercurials.

" In the latter stages of fever, on the contrary, if this affection of the liver still continue, which is not uncommon, either from its having been neglected in the early stage, or from its proving more obstinate than usual, I have always found the minute doses of blue pill above specified, given every six or eight hours, most beneficial. Combined, indeed, with moderate evacuations of blood from the part, or (when the tenderness is inconsiderable, and the affection of the liver rather betrays itself by a vitiated secretion of bile, than by inflammatory symptoms,) blisters applied to the region of this organ, they rarely fail to restore due action to it, unless the nature of the fever, or constitution of the patient, be very unfavourable; and thus often remove the fever, which, when its symptoms have become mild, is frequently at this period prolonged by the local affection alone.

" When the sympathetic disease, previous to the attack of fever, has chiefly affected other parts, the bowels, the lungs, the brain, &c. we still find the part most affected by that disease, suffering most in the fever; and the same plan of treatment, *mutatis mutandis*, is applicable, except that the same benefit is not to be expected from the specific operation of mercury.

" When indigestion has not arrived at its second stage at the time the fever makes its attack, the accession of this disease, by increasing the inflammatory tendency, often induces that stage. The vessels, although they had not been sufficiently weakened to yield to the usual force of the circulation, yield to its increased force; and it particularly deserves attention, that an attack of fever, as I have repeatedly witnessed, is often the means of permanently converting the first into the second stage of indigestion; so that the fever leaves behind it tenderness of the epigastrium, and more or less hardness of the pulse, where they had not previously existed.

" When this is the case to any considerable degree, the patient generally becomes liable to a renewal of fever from slight causes; and if the morbid state of the digestive organs is not removed, he is often exhausted by repeated attacks of fever, which, as the debility increases, assume a more chronic form, and often at length terminate in typhus, or the more severe species of what has been called nervous fever.

"Local congestion or inflammation, as might be expected, although none of the symptoms of indigestion have preceded, sometimes takes place in fever, that is, when the force of the circulation is morbidly increased. This is most apt to happen in the brain or liver.

The principle of treatment, as far as I have been able to observe, is still the same. In the latter case, however, the means of relief are generally sooner successful, and the patient bears them better. The treatment of fever, in those who have long laboured under the symptoms of indigestion, requires great circumspection. It is surprising after how moderate a degree of fever symptoms of danger often arise in them, and indeed death itself actually ensues. Both the vascular and nervous systems of some organ necessary to life have been previously enfeebled, and it wholly loses its power before the fever produces any very general effect. The patient dies as much of the disease under which he has so long laboured, as of the fever which has supervened on it, and that at a time perhaps when his physician's mind is fatally abstracted from the former. These observations have been so often impressed on me in the course of practice, that I cannot help, in a particular manner, calling the attention of others to them. The more we see of disease, we shall, I think, be the more ready to admit that the digestive organs form so important a part of the animal system, and are so intimately connected with every other part of it, that there is no case in which their state can with safety be disregarded." 311.

The rapid exhaustion of the first edition, (though not always an infallible criterion of superlative merit in a book) together with the very general expression of approbation which we hear from all quarters, sufficiently attest that Dr. Philip's work has passed the ordeal of criticism with triumph, and may now be ranked amongst the standard productions of the British press.

